# Cases of asphyxia in children and adolescents: a retrospective analysis of fatal accidents, suicides, and homicides from 1998 to 2017 in Hamburg, Germany

**DOI:** 10.1007/s00414-020-02248-6

**Published:** 2020-01-18

**Authors:** Dieu Phuong Mosek, Jan Peter Sperhake, Carolin Edler, Klaus Püschel, Ann Sophie Schröder

**Affiliations:** 1 Department of Internal Medicine, Wilhelmsburger Hospital Groß-Sand, Groß-Sand 3, 21107 Hamburg, Germany; 2 Department of Legal Medicine, University Medical Center Hamburg–Eppendorf, Butenfeld 34, 22529 Hamburg, Germany

**Keywords:** Asphyxia, Childhood deaths, Cause of death, Manner of death, Autopsy

## Abstract

**Purpose:**

Injury-related asphyxia is one of the most common causes of death in children in Germany. However, only a few systematic studies have analyzed the causes and circumstances of asphyxia in children and adolescents.

**Methods:**

All cases of asphyxia in children and adolescents (0–21 years of age) among the Hamburg Legal Medical Department’s autopsy cases from 1998 to 2017 were retrospectively analyzed with special focus on how often external findings were completely absent.

**Results:**

Among 249 cases of fatal asphyxia, 68% were accidents, 14% were suicides, and 13% were homicides. Most of the cases involved boys. Adolescents and young adults aged 15–21 years represented the main age group. Drowning was the leading mechanism of asphyxia. Younger age was associated with less frequent detection of external signs of asphyxia in the postmortem external examination. Petechial hemorrhages were the most common visible external indication of asphyxia. No external findings indicative of asphyxia were present in 14% of the cases.

**Conclusion:**

Asphyxia in children and adolescents often involves accidents. However, postmortem external examination alone is insufficient to identify asphyxia and the manner of death.

## Introduction

Asphyxia is the generic term for mechanisms that cause a deficiency of the tissue oxygen supply that is required to sustain metabolic function [1]. The four physiological causes of asphyxia are reduced oxygen in the environment, reduced blood oxygenation, reduced cardiovascular oxygen transfer, and interference with cellular oxygen absorption [2].

In the forensic context, the mechanisms of asphyxia include mechanical causes, such as strangulation, aspiration of foreign bodies or boluses, or constriction; changes in breathable air, such as flue gas inhalation; strangulation mechanisms, such as hanging or ligature strangulation; positional asphyxia; and drowning [3].

Asphyxia and acts of violence are the leading injury-related causes of death in children under 15 years of age in Germany [4]. Previous studies have primarily examined accidental deaths caused by asphyxia; few data are available on the specific distribution of deaths according to the causes of asphyxia [5]. The medicolegal specialist plays a particularly important role in preventing these cases of death by reconstructing the circumstances of death, analyzing the injury patterns, and identifying potential sources of danger [6].

Postmortem external examination alone does not reliably identify asphyxia. Indications of asphyxia, such as petechial hemorrhages or findings of injury that suggest the asphyxia mechanism, may be completely missing and/or misinterpreted by non-specialist postmortem examination physicians. An autopsy to clarify the cause of death should be performed in all suspicious cases despite the fact that a precise cause of death cannot be determined by autopsy in all cases. Diagnosing asphyxia in infants and small children is particularly difficult because violent death in children may leave almost no visible traces [2]. Particularly challenging is differentiating between death by asphyxia and sudden infant death syndrome (SIDS); such differentiation is not always possible [7].

The present study was a systematic overview of all cases of asphyxia in children, adolescents, and young adults in a German metropolitan area that involved a medicolegal autopsy between 1998 and 2017. Our findings present the spectrum of different mechanisms of asphyxia and the circumstances of death in various age groups, and the rates of diagnosis of asphyxia during the postmortem external examination.

## Materials and methods

All autopsies performed at the Department of Legal Medicine (Institut für Rechtsmedizin, IfR) of the University Medical Center Hamburg-Eppendorf from 1 January 1998 to 31 December 2017 were retrospectively analyzed. Cases in which the postmortem medical examiner had considered all circumstances for the diagnosis of fatal asphyxia in children, adolescents, or young adults aged 0–21 years were included in the study.

The available documents were descriptively analyzed regarding the circumstances of death, mechanisms of asphyxia, pre-existing medical conditions, hospital treatments, resuscitation, time and place of occurrence, presence of a chaperone, age, sex, and presence of asphyxia findings during the postmortem external examination. Microsoft Excel (Microsoft Corp., Redmond, WA, USA) was used for data input and analysis.

## Results

The IfR performed 23,638 autopsies from 1998 to 2017. Death caused by asphyxia was identified in 249 autopsies (1% of all autopsies; 242 medicolegal autopsies and 7 private autopsies) in children and adolescents (0–21 years of age).

### Circumstances of death (accident, suicide, or homicide)

Table 1 shows the circumstances of death (accident, suicide, or homicide) according to age group. Most cases involved accidents (more than half of the cases of death in all age groups). Suicides and homicides occurred with almost equal frequency. Suicides occurred in only one case at the age of 9 years and then in all ages from 12 to 21 years of age. The most common suicide mechanism was hanging. Homicides occurred most often in newborns.

**Table 1:** Circumstances of death (accident, suicide, or homicide) according to age group

Age groups	Accident	Suicide	Homicide	Unclear	Total
Newborns (0–4 weeks)	13 (68%)	0 (0%)	6 (32%)	0 (0%)	19
Infants (> 4 weeks–1 year)	15 (71%)	0 (0%)	2 (10%)	4 (19%)	21
Toddlers (1–4 years)	46 (88%)	0 (0%)	5 (10%)	1 (2%)	52
Schoolchildren (5–14 years)	36 (64%)	6 (11%)	9 (16%)	5 (9%)	56
Adolescents and young adults (15–21 years)	58 (57%)	30 (30%)	10 (10%)	3 (3%)	101
Total	168 (68%)	36 (14%)	32 (13%)	13 (5%)	249

### Mechanisms of asphyxia

Table 2 shows the distributions of age and sex among the asphyxia mechanisms, which are divided into drowning, gas inhalation, aspiration of endogenous fluids, aspiration of foreign objects, smothering of the respiratory tract, suffocation during birth, positional asphyxia and constriction, hanging, manual strangulation, ligature strangulation, other strangulation mechanisms, and combined mechanisms of asphyxia. Two-thirds of the cases of death caused by asphyxia involved boys, most of whom had died from accidents. Girls were more often the victims of homicides.

**Table 2:** Mechanisms of asphyxia and circumstances of death according to age and sex

Mechanism of asphyxia in different age groups	Drowning		Suicide		Homicide		Unclear		Total
	m	f	m	f	m	f	m	f	
Drowning									
Newborns	1	0	0	0	0	1	0	0	2
Infants	1	2	0	0	0	0	0	0	3
Toddlers	19	5	0	0	1	0	0	1	26
Schoolchildren	10	3	0	0	0	1	1	0	15
Adolescents and young adults	24	6	3	1	0	0	1	1	36
Sum	55	16	3	1	1	2	2	2	
Total	71		4		3		4		82
Gas inhalation									
Newborns	1	1	0	0	0	0	0	0	2
Infants	5	5	0	0	0	0	0	0	10
Schoolchildren	7	3	0	0	0	0	2	0	12
Adolescents and young adults	7	3	1	0	0	0	0	0	11
Sum	20	12	1	0	0	0	2	0	
Total	32		1		0		2		35
Aspiration of endogenous fluids									
Newborns	0	1	0	0	0	0	0	0	1
Infants	1	3	0	0	0	0	0	0	4
Toddlers	4	0	0	0	2	0	0	0	6
Schoolchildren	4	5	0	0	0	0	0	0	9
Adolescents and young adults	8	3	1	3	0	0	0	0	15
Sum	17	12	1	3	2	0	0	0	
Total	29		4		2		0		35
Hanging									
Toddlers	0	1	0	0	0	0	0	0	1
Schoolchildren	0	0	5	1	0	0	1	1	8
Adolescents and young adults	0	0	15	5	0	0	1	0	21
Sum	0	1	20	6	0	0	2	1	
Total	1		26		0		3		30
Combination of mechanisms of asphyxia									
Infants	1	0	0	0	1	1	1	0	4
Toddlers	1	0	0	0	0	0	0	0	1
Schoolchildren	0	1	0	0	1	1	0	0	3
Adolescents and young adults	1	0	0	0	0	3	0	0	4
Sum	3	1	0	0	2	5	1	0	
Total	4		0		7		1		12
Smothering of respiratory tract									
Newborns	0	0	0	0	1	2	0	0	3
Infants	1	0	0	0	0	0	2	1	4
Toddlers	0	0	0	0	1	0	0	0	1
Schoolchildren	0	0	0	0	1	0	0	0	1
Adolescents and young adults	0	0	1	0	0	0	0	0	1
Sum	1	0	1	0	3	2	2	1	
Total	1		1		5		3		10
Asphyxia during birth									
Newborns	7	3	0	0	0	0	0	0	10
Total	10		0		0		0		10
Manual strangulation									
Newborns	0	0	0	0	0	2	0	0	2
Schoolchildren	0	0	0	0	3	0	0	0	3
Adolescents and young adults	0	0	0	0	1	4	0	0	5
Sum	0	0	0	0	4	6	0	0	
Total	0		0		10		0		10
Positional asphyxia and constriction									
Newborns	0	1	0	0	0	0	0	0	1
Infants	3	0	0	0	0	0	0	0	3
Toddlers	1	0	0	0	0	0	0	0	1
Schoolchildren	2	0	0	0	0	0	0	0	2
Adolescents and young adults	2	0	0	0	0	0	0	0	2
Sum	8	1	0	0	0	0	0	0	
Total	9		0		0		0		9
Aspiration of foreign objects and boluses									
Infants	0	1	0	0	0	0	0	0	1
Toddlers	3	0	0	0	0	0	0	0	3
Schoolchildren	1	0	0	0	0	0	0	0	1
Adolescents and young adults	2	0	0	0	0	0	0	0	2
Sum	6	1	0	0	0	0	0	0	
Total	7		0		0		0		7
Ligature strangulation									
Toddlers	1	0	0	0	0	1	0	0	2
Schoolchildren	0	0	0	0	0	2	0	0	2
Adolescents and young adults	0	0	0	0	1	1	0	0	2
Sum	1	0	0	0	1	4	0	0	
Total	1		0		5		0		6
Other mechanisms of strangulation									
Toddlers	0	1	0	0	0	0	0	0	1
Adolescents and young adults	1	1	0	0	0	0	0	0	2
Sum	0	2	0	0	0	0	0	0	
Total	3		0		0		0		3
Sum total	119	49	26	10	13	19	9	4	
Grand total	168		36		32		13		249

### Pre-existing medical conditions, hospital treatment, and resuscitation

Fifty-four cases (22%) had experienced a chronic medical condition and 22 (9%) had experienced an acute medical condition. Six cases (2%) had undergone a surgical intervention within the previous 2 weeks.

Additionally, a hospital treatment was administered following the incident in 39 cases (16%). Medical personnel had performed resuscitation in 95 cases (38%).

### Time of occurrence

In total, 116 (47%) cases of asphyxia had occurred during the night (6:00 pm–6:00 am), 89 (36%) had occurred during the day (6:00 am–6:00 pm), and the time of occurrence remained unknown in 44 cases (17%). Accidents had occurred almost equally during the day and night.

### Place of occurrence

A total of 128 (51%) cases of asphyxia had occurred at home, 95 (38%) in public, 13 (5%) in care facilities, and 9 (4%) in hospitals. The home of the offender had been the place of occurrence in two cases (1%), and the place of occurrence remained unknown in two cases (1%).

### Chaperone

A chaperone had either been in the same building or within shouting distance in 132 cases (53%). The chaperone was either an adult family member or supervising staff of the respective institution (teacher, childcare worker, nursing staff, or correctional facility staff). Thus, the chaperone was also the offender in homicides. In 107 cases (43%), the incident had occurred without a chaperone close by. The presence of a chaperone could not be ascertained in 10 cases (4%). A chaperone had been present in close proximity in 97 (58%) accident-related cases of death and not in close proximity in 64 cases (38%). A chaperone had been in the same building or within shouting distance in 11 of the suicide cases (31%).

### Findings of asphyxia in the postmortem external examination

Neither external indications of asphyxia (e.g., petechial hemorrhages, facial edema, or cyanosis) nor the mechanism of asphyxia (e.g., spotty, recessed livor mortis; injuries; or hematomas) were identified in 35 cases (14%) (Table 3). Fewer indications of asphyxia were found during the postmortem external examinations of younger children. Petechial hemorrhages were present in 92 cases (37%): in 46 cases (27%) of accidents, 18 cases (50%) of suicides, 23 cases (72%) of homicides, and in 5 cases (38%) of the deaths where the circumstance of death remained unclear. Petechial hemorrhages were found in 32 (34%) cases with cardiopulmonary resuscitation (CPR) and in 60 (39%) cases without CPR (Table 4).

**Table 3:** Findings of asphyxia during the postmortem external examination in different age groups

Age groups	Indications of asphyxia in postmortem external examination
Newborns	11 (58%)
Infants	15 (71%)
Toddlers	46 (88%)
Schoolchildren	50 (89%)
Adolescents and young adults	92 (91%)
Total	214 (86%)

**Table 4:** Findings of petechial hemorrhages compared with CPR and non-CPR regarding the different categories of death

Circumstance of death	Professional CPR	
	No (*n* = 154)	Yes (*n* = 95)
	Findings of petechial hemorrhages	Findings of petechial hemorrhages
Accident	20	26
Suicide	13	5
Homicide	23	0
Unclear	4	1
Total	60 (39%)	32 (34%)

### Drowning

**Fig. 1:**
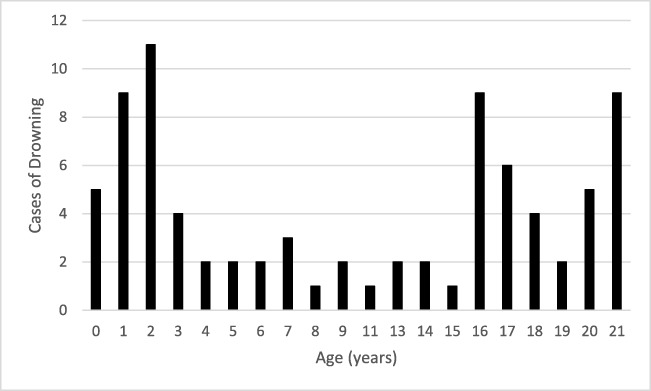
Cases of drowning by age

The leading mechanism of asphyxia was drowning (82 cases, 33%). The most prevalent ages among cases of drowning were 1, 2, 16, and 21 years (Fig. 1).

A chaperone had been present in 32 cases (39%). Twenty-three children (28%) had known how to swim, 34 (41%) had been non-swimmers, and no information was available in 24 cases (29%). Most cases of drowning occurred in public bodies of water (64 cases, 78%). The postmortem external examination revealed indications of drowning in 71 cases (87%), and a “mushroom of foam” adjacent to the mouth and nose was present in 14 cases (17%).

### Gas inhalation

Gas inhalation was the mechanism of asphyxia in 35 cases (14%). There was one case of hydrogen sulfite intoxication; all other cases involved carbon monoxide intoxication. The postmortem external examination showed clear indications of gas inhalation in 34 cases (97%). Light red livor mortis and airway soot deposition were the most common findings, identified in 29 cases (83%).

### Aspiration of endogenous fluids

Aspiration of endogenous fluids was the mechanism of asphyxia in 35 cases (14%). The injuries sustained in two homicides and four suicides resulted in aspiration of blood. Twenty cases (57%) had occurred at night and 18 (51%) had occurred at home. A chaperone had been present in 19 cases (54%). Almost half of the aspiration-related cases of death (16 cases, 46%) involved individuals with pre-existing medical conditions. The postmortem external examination showed indications of asphyxia in 29 cases (83%).

### Hanging

Death by hanging occurred in 30 cases (12%); 87% of the cases were suicides, 1 case (3%) was an accidental hanging, and in 3 cases (10%), the cause of the hanging remained unclear. Most of the cases had occurred during the night (19 cases, 63%). Only children aged ≥ 9 years were involved in death by hanging. Twenty cases (67%) had occurred at home. The postmortem external examination revealed indications of asphyxia in 28 cases (93%); petechial hemorrhages were found in 20 cases (67%), and strangulation marks were found in 26 (87%). Laryngeal or hyoid bone fractures were identified in four cases (13%) at the ages of 16, 17 (2 cases), and 21 years.

### Combined mechanisms of asphyxia

The following combinations of asphyxia mechanisms occurred among 12 cases (5%): mechanical and ligature strangulation (*n* = 2), neck compression and smothering of the respiratory tract (*n* = 2), constriction and smothering of the respiratory tract (*n* = 2), drowning and carbon monoxide poisoning (*n* = 2), drowning and manual strangulation (*n* = 1), drowning and smothering of the respiratory tracts (*n* = 1), aspiration of endogenous fluids and manual strangulation (*n* = 1), and neck and chest compression (*n* = 1).

### Smothering of the respiratory tract

Smothering of the respiratory tract was the mechanism of asphyxia in 10 cases (4%). One case was a suicide in which a 21-year-old man had suffocated himself by putting a plastic bag over his head. Another case involved the accidental death of a 10-month-old child under a bed cover while asleep. Indications of asphyxia were found during the postmortem external examination in seven cases (70%), and most were in the form of petechial hemorrhages (4 cases, 40%).

### Asphyxia during birth

Death of newborns secondary to asphyxia during birth occurred in 10 cases (4%). Five cases (50%) occurred at the hospital, four (40%) occurred at home, and one (10%) occurred at a birthing house. Resuscitation measures were performed in five cases (50%). The postmortem external examination revealed indications of asphyxia in three of the children (30%).

### Manual strangulation

Death solely by manual strangulation occurred in 10 cases (4%). Nine cases (90%) showed strangulation marks and nine (90%) showed petechial hemorrhages.

### Positional asphyxia and constriction

Positional asphyxia and constriction was the mechanism of asphyxia in nine cases (4%). All involved accidents. Externally visible indications of asphyxia were identified in eight cases (89%).

### Aspiration of foreign objects

Aspiration was the mechanism of asphyxia in seven cases (3%), all of which involved accidents. Resuscitation measures had been performed in four cases (57%). The postmortem external examination revealed signs of asphyxia in six cases (86%); petechial hemorrhages were the most frequently found sign (4 cases, 57%).

### Ligature strangulation

Ligature strangulation occurred in six cases (2%), and postmortem external examination showed findings of asphyxia in all cases.

### Other mechanisms of asphyxia

**Fig. 2:**
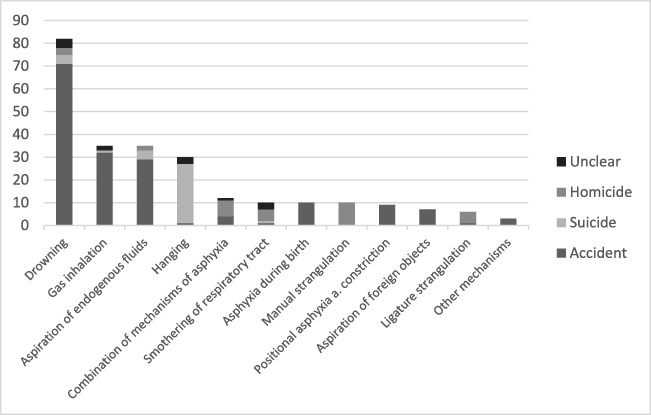
Causes and categories of asphyxia

The following additional mechanisms of asphyxia occurred (3 cases, 1%): dislocation of a tracheal cannula at home (pre-existing medical condition: respiratory distress syndrome), status asthmaticus, and complication during tracheotomy at the hospital (pre-existing medical condition: Duchenne muscular dystrophy with the development of acute pneumonia). The different causes and categories of asphyxia are summarized in Fig. 2.

## Discussion

Asphyxia in children and adolescents is a rare occurrence when comparing the total number of autopsies with the total number of child deaths reported by the German Federal Statistical Office. In the City-state of Hamburg alone, 126 children aged 0–20 years died in 1998 (19,228 total deaths), and 107 children died in 2016 (17,267 total deaths) [8]. However, asphyxia-related deaths are mostly avoidable cases of death and can be averted with appropriate preventive measures [9–11].

The most common causes of asphyxia in this analysis were accidents, and most involved boys. Suicides involved children from school age onward. Boys were also more prevalent in suicides, whereas girls were more often involved in homicides. Hanging was the leading method of suicide. The largest number of asphyxia cases involved adolescents and young adults aged 15–21 years, and most incidents occurred at home with the exception of drowning, which occurred predominantly in public bodies of water. This distribution is consistent with previous publications in studies of non-natural child deaths in Estonia, Canada, Sri Lanka, Turkey, and the USA [12–16].

In our study, deaths by asphyxia occurred during both day and night. In 1993, a study by Flobecker et al. [17] revealed an increase in accident-related asphyxia deaths in children in Sweden during the early morning hours and in the afternoon. Additionally, the literature shows seasonal increases for specific types of asphyxia. In 2006, Azmak [18] evaluated asphyxia-related deaths in all age groups in Turkey and found that most hangings occurred during spring and summer, and that most carbon monoxide poisonings occurred during winter. However, in 2014, Bhosle et al. [19] examined hanging in adolescents and found an increased incidence from 2006 to 2009 for no apparent reason. Time of day and seasonal variation in the exposure rate have been shown to play roles in drowning. In two recent Australian studies, Peden et al. [20, 21] identified increased drowning occurrences in children during school vacations and in the bathtub during bathing, from 4:00 pm–6:00 pm.

In 2018, a study by Sasso et al. [22] of asphyxia-related injuries in urgent care facilities in the USA revealed chronic pre-existing medical conditions in 72.8% of the patients. Patients with a pre-existing medical condition also had a higher risk of dying from an asphyxia-related incident [22]. Pre-existing medical conditions did not play a major role as a concomitant circumstance in cases of asphyxia in children. Predominantly, healthy children were involved in our study, and only those who died by aspiration of endogenous fluids showed an increased incidence of pre-existing medical conditions. This is to be expected because aspiration usually only occurs secondary to other physical impairments.

Our analysis showed that petechial hemorrhages were the most common externally visible finding indicating asphyxia. These appeared most often in children who had died of ligature strangulation, manual strangulation, or hanging. The presence of petechial hemorrhages in cases of strangulation differs vastly in the literature. A 2016 study by Ma et al. [23] of cases of hanging and strangulation in all age categories in China showed that petechial hemorrhages were present in 95% of hanging cases and in 52% of strangulation cases. However, in a 2016 study of hanging in Italy, Russo et al. [24] found only conjunctival petechial hemorrhages in 11% of the cases.

Petechial hemorrhages are caused by venous obstruction in continuous arterial flow. Other than asphyxia, such hemorrhages can be caused by many other conditions such as asthma attacks, status epilepticus, coughing, vomiting, and cardiac massage. Therefore, their sole presence is not clear evidence of asphyxia [25–27]. However, when present in conjunction with other findings of asphyxia detected during the autopsy, petechial hemorrhages are useful for distinguishing asphyxia-related death from SIDS [28]. In our study, there was no higher incidence of petechiae in cases with CPR.

The findings related to asphyxia gathered during autopsy, such as hyperinflated lungs, bleeding beneath organ membranes, and blood stasis in the organs, are also nonspecific. In 2010, Fracasso et al. [29] found hemorrhages causing asphyxia beneath the organ membranes of newborns who had died of asphyxia as well as in newborns who had died of SIDS, sepsis, or respiratory tract infections. Findings of asphyxia may also be completely missing when the attacker has significantly more physical strength than the victim [30].

Our results regarding the verification of asphyxia and the circumstances of death indicate that a postmortem external examination by itself is insufficient. In addition to autopsy and evaluating pre-existing conditions, histological, toxicological, and neuropathological examinations are also useful to achieve the correct diagnosis [31, 32]. However, even with the most meticulous execution of all of these examinations, it is not always possible to clearly differentiate between an asphyxia-related homicide with very few traces and death from internal causes (e.g., SIDS).

This study has several limitations because of its retrospective design. Because the data were gathered from autopsy reports, they are not completely standardized and, thus, are dependent on the relative quality of the examination and documentation. As described by Colville-Ebeling et al. [33] in 2013, the quality of the data is largely dependent on the experience of the medical examiner. The data are not adequate to shed light on a potential dark field of undetected asphyxia-related cases of death (e.g., external signs of asphyxia are present but no autopsy is performed; however, in our study region, it is highly unlikely that such cases do not undergo a medicolegal autopsy). Because the diagnosis of death caused by asphyxia is sometimes based on external findings, circular reasoning cannot be totally excluded. For example, if injuries on the skin of the neck and petechial hemorrhages on the conjunctiva have led to a diagnosis of manual strangulation, ligature strangulation, or hanging, these diagnoses will be found on a regular basis in cases of this type of death.

In the present study, lack of information or only estimated values were often obtained regarding, for example, the time of occurrence or time of death, and regarding the children’s ability to swim. Regarding the presence of a chaperone, we included cases in which a chaperone had been in the same building or within shouting distance, as had occurred in care facilities such as childcare facilities or schools. However, when a chaperone is present, only direct supervision is a true preventive factor [9, 17].

Finally, the statistical power of our data is limited by the relatively small number of cases. However, the specific objective of this study was individual case assessment and systematic presentation to facilitate categorizing cases into accidents, suicides, or homicides. This is the only way to identify hazardous situations and provide recommendations for preventive measures.
